# Response to the Combination of Osimertinib, Dabrafenib, and Trametinib in Leptomeningitis From *EGFR*-Mutant NSCLC With Acquired *BRAF* V600E Mutation: A Case Report

**DOI:** 10.1016/j.jtocrr.2021.100192

**Published:** 2021-05-20

**Authors:** Orion Valet, Aurélie Swalduz, Maxime Boussageon, Adrien Buisson, Virginie Avrillon, Bénédicte Mastroïanni, Maurice Pérol

**Affiliations:** a Department of Medical Oncology, Centre Léon Bérard, Lyon, France; b Department of Biopathology, Centre Léon Bérard, Lyon, France

**Keywords:** Case report, *EGFR* mechanism of resistance, *BRAF* mutation, Dabrafenib-trametinib combination, Leptomeningeal disease

## Abstract

Molecular sequencing after highly potent targeted gene inhibitors have suggested resistant tumors can display substantial heterogeneity. Among these various mechanisms of resistance, secondary mutations on targetable oncogenes have been identified. *BRAF* V600E, as a bypass mechanism on disease progression while receiving osimertinib therapy, has been reported in 3% of *EGFR-*mutated patients. Few case reports described the efficacy of the association of osimertinib and dabrafenib plus trametinib. Here, we report, for the first time, a case of a patient treated with this association, with a prolonged response on leptomeningeal metastasis. We also provide a comprehensive overview of the available literature on the efficacy and tolerance of this association.

## Introduction

Osimertinib is a third-generation EGFR tyrosine kinase inhibitor (TKI) active in the central nervous system and is the current standard of care for the first-line treatment of *EGFR*-mutant NSCLC.^1^ Despite an improvement in progression-free survival (PFS) and overall survival in comparison with first-generation EGFR TKIs (median PFS of 18.9 mo), patients invariably experience disease progression characterized by heterogeneous mechanisms of resistance including 3% of *BRAF* V600E bypass mutations. The combination of the BRAF inhibitor dabrafenib and MEK inhibitor trametinib is a standard therapeutic option for patients with NSCLC harboring a primary *BRAF* V600E mutation.^2^

Here, we report the case of a complete leptomeningeal response to the association of osimertinib, dabrafenib, and trametinib (O+D+T) in a patient with *EGFR*-mutant NSCLC harboring a *BRAF* V600E mutation as a resistance mechanism to osimertinib.

## Case Presentation

**Figure 1:**
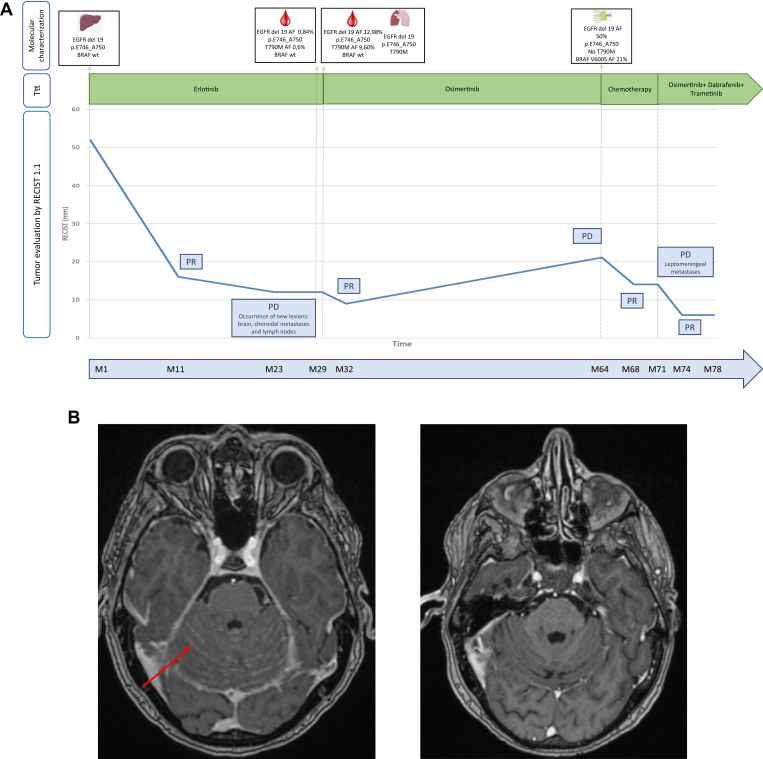
*(A)* Timeline of the patient’s clinical, radiologic, molecular course, and treatment. *(B)* The red arrow is pointing out the leptomeningeal contrast enhancement, confirming the infratentorial leptomeningeal metastatic involvement that disappeared after 3 months of dabrafenib-trametinib and osimertinib combination. AF, allelic fraction; del, deletion; PD, progression disease; PR, partial response; RECIST, Response Evaluation Criteria in Solid Tumors; Ttt, treatment; wt, wild type.

**Figure 2:**
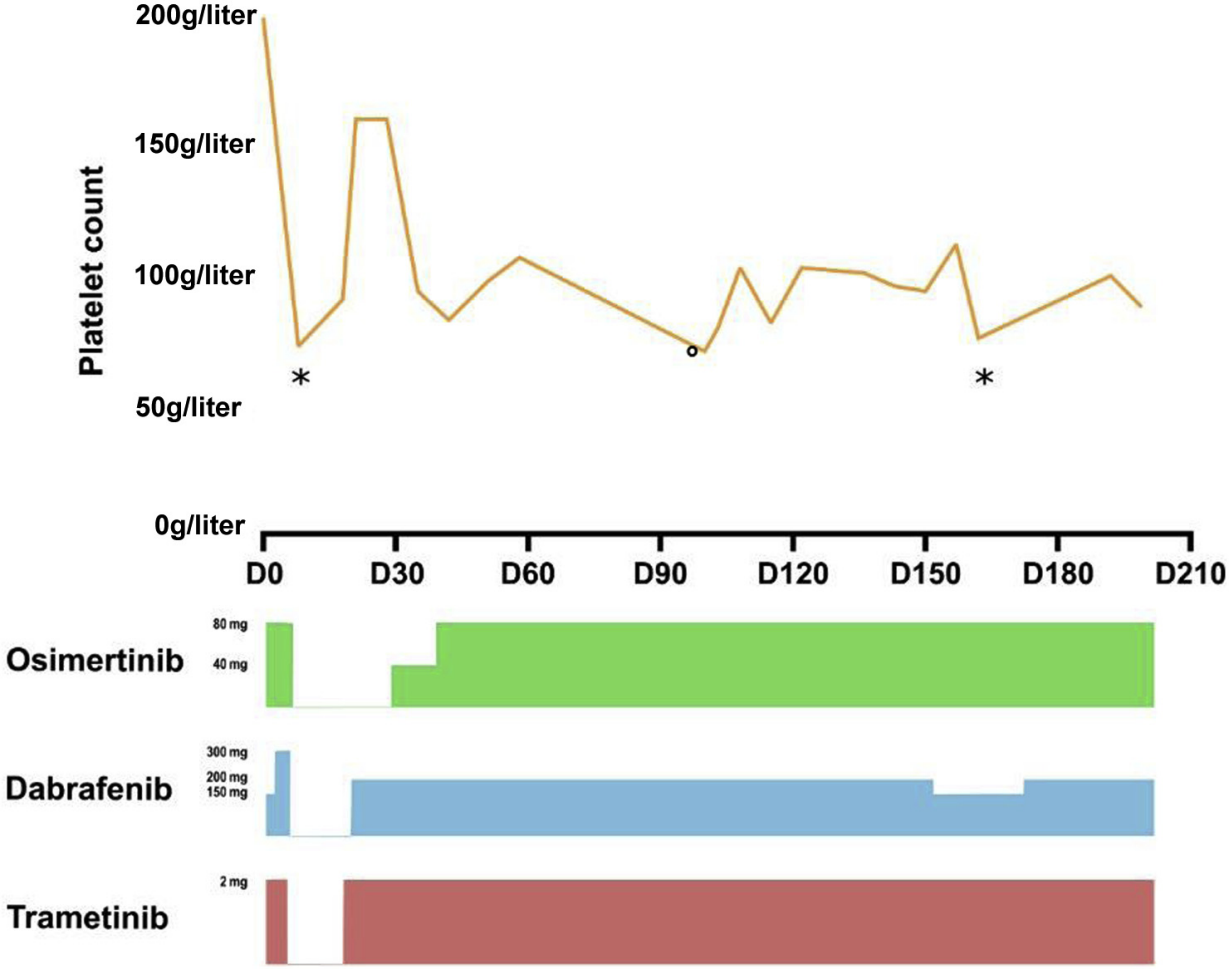
Adverse events and dose reductions. ∗ Pyrexia. ° Pneumonitis. D, day.

A 67-year-old never-smoker man presented vertebral pain caused by a multisite metastatic lung adenocarcinoma (bone, liver, lymph nodes, and brain), harboring an *EGFR* exon 19 deletions (p.E746_A750del) with asymptomatic infratentorial brain metastases without clinical and radiologic signs of meningitis. Molecular results and panels performed at each time point are described in the Appendix (Supplementary data). He received erlotinib at 150 mg/day with a good initial response (Fig. 1*A*). After 26 months, he experienced vision alterations with metamorphopsia, which was found to be choroid metastases without any sign of leptomeningeal disease on brain magnetic resonance imaging (MRI). Bronchial and liquid biopsies identified an *EGFR* p.T790M mutation and the initial *EGFR* exon 19 deletion with no *BRAF* mutation, and the patient was subsequently treated with osimertinib leading to an almost complete response. After 3 years of osimertinib, he experienced progressive disease, and next-generation sequencing performed on a lymph node core needle biopsy revealed an *EGFR* exon 19 deletion, the loss of T790M mutation, and the appearance of the *BRAF* V600E mutation with a high allelic fraction (21%). Chemotherapy with carboplatin and pemetrexed was initiated with a partial response; but 6 months later during maintenance therapy with pemetrexed, he experienced neurologic symptoms including confusion, walking disability, vomiting, neck stiffness, and diplopia, and a brain MRI confirmed the existence of carcinomatous meningitis. On the basis of the previous detection of the *BRAF* V600E mutation, after obtaining patient informed consent, a combination of O+D+T at full dose was initiated. An early and dramatic response was observed with complete regression of all clinical neurologic manifestations and the disappearance of signs of meningitis on the first brain MRI at 3 months (Fig. 1*B*), allowing the patient to regain full autonomy. No subsequent extracranial progression was observed, and response is still ongoing after 12 months of treatment. However, several treatment-related toxicities occurred (Fig. 2). First, grade 2 pyrexia was observed at day 5 after treatment initiation; dabrafenib was discontinued with prompt fever improvement, then reintroduced in a reduced dose at 100 mg twice a day. Recurrence of pyrexia after 4 months led to another temporary reduction in the dose of dabrafenib to 75 mg for 1 week, before reescalation to 100 mg twice a day. Grade 2 thrombocytopenia was also observed, leading to the discontinuation of the three TKIs for 2 weeks with gradual reintroduction. The platelet count then remained stable between 80 g/liter and 100 g/liter without hemorrhagic symptoms. Finally, grade 1 interstitial pneumonitis was detected on a computed tomography scan after 3 months of treatment. Considering the clinical improvement of neurologic symptoms, the combination was not discontinued and prednisolone at a dose of 40 mg/day was introduced. A follow-up computed tomography scan did not reveal any improvement or worsening of the pneumonitis and the patient still receives prednisolone at the dose of 20 mg/day without respiratory symptoms.

## Discussion

Understanding the mechanisms of acquired resistance to osimertinib is still an area of active investigation. A large panel of on-target and bypass mechanisms have been described under osimertinib However, management of osimertinib postprogression remains unclear. The identification of targetable mechanisms of resistance has exposed combining TKIs as an interesting strategy. It has already been evaluated for patients harboring *MET* amplification as a mechanism of resistance with promising results and a favorable profile of tolerance. RET inhibition associated with osimertinib also revealed encouraging results in the case reports of patients with *RET*-fusion.

The *BRAF* V600E mutation as a resistance mechanism to osimertinib is present in 3% of patients. Previously reported cases revealed O+D+T efficacy, with some rapid and prolonged responses (Table 1) but none of them presented leptomeningeal metastases. In our case, *BRAF* mutation was identified at disease progression with osimertinib, but because chemotherapy was the standard of care, the O+D+T combination was not immediately introduced after resistance to osimertinib. This treatment sequence may have modified the subclonal heterogeneity of the tumor and resensitized it to EGFR inhibition, by prompting the proliferation of the sensitive *EGFR*-mutated clone after removing the selective pressure from the EGFR TKI.^3^ However, experience gained from patients rechallenged with first-generation EGFR TKIs reflects that, despite an interesting initial disease control rate (45%), the median PFS remains short (around 2.0 mo).^4^ The relative long-term response (at least 1 y) achieved using O+D+T argues in favor of the efficacy of this treatment combination.

**Table 1:** Case Reports of Patients Harboring Concomitant EGFR Mutations and BRAF V600E Treated With TKI Combination in Previously Reported Cases

Authors and DOI	Sex	Smoking History	*EGFR* Mutation	*BRAF* Alteration	Additional Mutation	Treatment and Dose	Line	Chemotherapy Between Resistance to TKI and Combination	Systemic Best Response	CNS Best Response	Duration of Treatment (Months)	Toxicity	Management of Toxicity
Ribeiro et al. 10.1038/s41698-021-00149-4	M	0	Del 19 T790M	V600E mutation	PIK3CA E54 5K	D 75 mg twice daily T 1 mg daily O 80 mg daily	4	Yes	PR	No metastasis	8	G1 fatigue G1 dysgueusia G1 pyrexia G1 nausea	Symptomatic treatment
Meng et al. 10.1016/j.lungcan.2020.05.036	F	0	Del 19 loss of T790M	V600E mutation	0	O dose NR D dose NR T dose NR	3	No	NR	No response	1.1	Pneumonitis	Stop treatment
Meng et al. 10.1016/j.lungcan.2020.05.036	M	0	Del 19 T790M	V600E mutation	0	O dose NR D dose NR T dose NR	3	No	PR	No metastasis	13.4	G2 pyrexia G2 nausea G2 vomiting	Dose reduction: D 50 mg twice daily T 0.5 mg daily O 80 mg daily
Zhou et al. 10.1016.j.lungcan.2019.10.014	M	0	L858R T790M	V600E mutation	0	O 80 mg daily D 150 mg twice daily T 2 mg daily	5	Yes	PR	No metastasis	2+	G2 rash G2 anorexia	None
Huang et al. 10.1016/j.jtho.2019.05.040	M	Former smoker	Del 19 T790M	V600E mutation	0	O 80 mg daily D 150 mg twice daily T 1 mg daily	3	No	SD	No metastasis	7.4+	G1 diarrhea paronychia	None
Xie et al. 10.1016/j.cllc.2020.06.008	F	0	L858R T790M	V600E mutation	TP53	O 80 mg daily V 960 mg daily	3	No	PR	PR	2	G4 vomiting	Dose reduction of V to 240 mg twice daily then discontinuation of treatment
Xie et al. 10.1016/j.cllc.2020.06.008	M	10 y	Del 19 T790M	V600E mutation	0	O dose NR V 480 twice daily	3	No	SD	No metastasis	4.5	None	Stop treatment
Dagogo-Jack et al. 10.1016/jtho.2019.05.046	F	0	Del 19	AGK-BRAF fusion	0	O 40 the 80 mg daily T 1 mg daily	5	Yes	PR	No metastasis	5	G2 diarrhea G2 fatigue G1 rash G1 digestive bleeding Colonic perforation	
Current case	M	0	Del 19 Loss of T790M	V600E mutation	0	O 80 mg daily D 150 mg twice daily T 2 mg daily	4	Yes	PR	CR	12+	G2 thrombocytopenia G2 pyrexia G1 pneumonitis	Dose reduction for thrombocytopenia and pyrexia corticosteroids for pneumonitis

CNS, central nervous system; CR, complete response; D, dabrafenib; Del, deletion; F, female; G, grade; M, male; NR, nonreported; O, osimertinib; PR, partial response; SD, stable disease; T, trametinib; TKI, tyrosine kinase inhibitor; V, vemurafenib.

Another major consideration when combining TKIs is the management of toxicity. In previous reports, the combination of O+D+T has mainly been associated with expected grade 1 to 2 toxicities including pyrexia, fatigue, and gastrointestinal disorders (Table 1) with no major signs of additional toxicities. In our case, pyrexia led to treatment interruption and dose reduction. Pyrexia is one of the toxicities often observed with BRAF inhibitors (6% of grade ≥3), but its severity seems to be enhanced when combined with MEK inhibitors. It often occurs during the ﬁrst month of treatment and prompt interruption of TKIs usually results in rapid resolution of symptoms. Most of the time, both drugs can be safely restarted at the same dose.^5^ Thrombocytopenia has been reported with osimertinib monotherapy but not with dabrafenib-trametinib and could worsen with the combination of three TKIs. Finally, pneumonitis can be observed with both osimertinib and dabrafenib-trametinib and could be a major issue for their combination and should be strictly monitored.

## Conclusion

The use of BRAF inhibitors combined with osimertinib for patients who experience disease progression with the *BRAF* V600 mutation seems to be feasible with interesting central nervous system efficacy even on leptomeningeal metastases. Acceptable overlapping toxicity was observed but close monitoring of symptoms of the patient is crucial especially pneumonitis-related symptoms. This case report also underscores the need for a repeated tumor or liquid biopsies to identify targetable mechanisms of resistance after progression with osimertinib.

## CRediT Authorship Contribution Statement

**Orion Valet:** Writing original draft.

**Aurélie Swalduz:** Writing review-editing, Conceptualization ideas.

**Maxime Boussageon:** Validation.

**Adrien Buisson:** Molecular analysis.

**Virginie Avrillon and Bénédicte Mastroianni:** Validation.

**Maurice Perol:** Writing review-editing, Conceptualization ideas, Resources.
