# Supplementary material for: Response to the Combination of Osimertinib, Dabrafenib, and Trametinib in Leptomeningitis From EGFR-Mutant NSCLC With Acquired BRAF V600E Mutation: A Case Report
Source: JTO Clin Res Rep. 2021 May 20;2(6):100192. doi: 10.1016/j.jtocrr.2021.100192 (PMC8474423; doi:10.1016/j.jtocrr.2021.100192)
Supplement: Supplementary Data [file mmc1.docx]

Appendix**: Molecular characterization and panel used**

Initial liver biopsy:

Sanger sequencing of EGFR (exon 18 to 21), *KRAS* (exon 2 and 3) and *BRAF (exon 15)* genes ***EGFR* del19 p.E746_A750, BRAF wt, KRAS wt**

Cell-free DNA under treatment with erlotinib before RECIST1.1 progression:

InvisionFirst® molecular panel

***EGFR* del 19 p.E746_A750 (AF 0.84%) p.T790M (AF 0.6%)**

***BRAF* wt**

Lung biopsy at progression with erlotinib:

Sanger sequencing of *EGFR* gene (exon 19 and 20)

***EGFR* del19 p.E746_A750, pT790M**

Cell-free DNA at progression with erlotinib:

InvisionFirst® molecular panel

***EGFR* del 19 p.E746_A750 (AF 12.98%) p.T790M (AF 9.60%)**

***BRAF* wt**

Lymph node biopsy:

NGS, kit FusionPlexRNA CTL (Archedex)

***EGFR* del 19 p.E746_A750 (AF 50%), No p.T790M**

***BRAF* V600E (AF 21%)**

InvisionFirst® molecular panel

InVisionFirst utilizes a proprietary enhanced tagged-amplicon sequencing (eTAm-Seq) method which allows for deep sequencing of genomic regions spanning thousands of bases from individual copies of fragmented ctDNA isolated from a peripheral blood sample. The panel is designed to identify genetic mutations that are known to be somatically altered in human malignancies by analyzing the plasma that is derived from a patient blood sample. This panel assesses either whole genes or select regions of interest (“hotspots”) within well-known, cancer-related genes that are validated targets for therapy, indicators for resistance to therapy, used as entry or exclusion criteria for clinical trials, markers of disease prognosis, and/or potential drivers of cancer based on published literature.

Single nucleotide variants and small insertions and deletions (InDels) are detected in all cited gene regions except FGFR1 (copy number changes only) and RET (fusions only). EGFR, ERBB2, FGFR1, and MET amplification are determined for all samples. ALK, NTRK1, RET and ROS1 fusion variants are identified for non-small cell lung cancer specimens. There is extensive exon coverage for PTEN (67.5%), TP53 (100%), STK11 (90.5%), and

CDKN2A (94%).


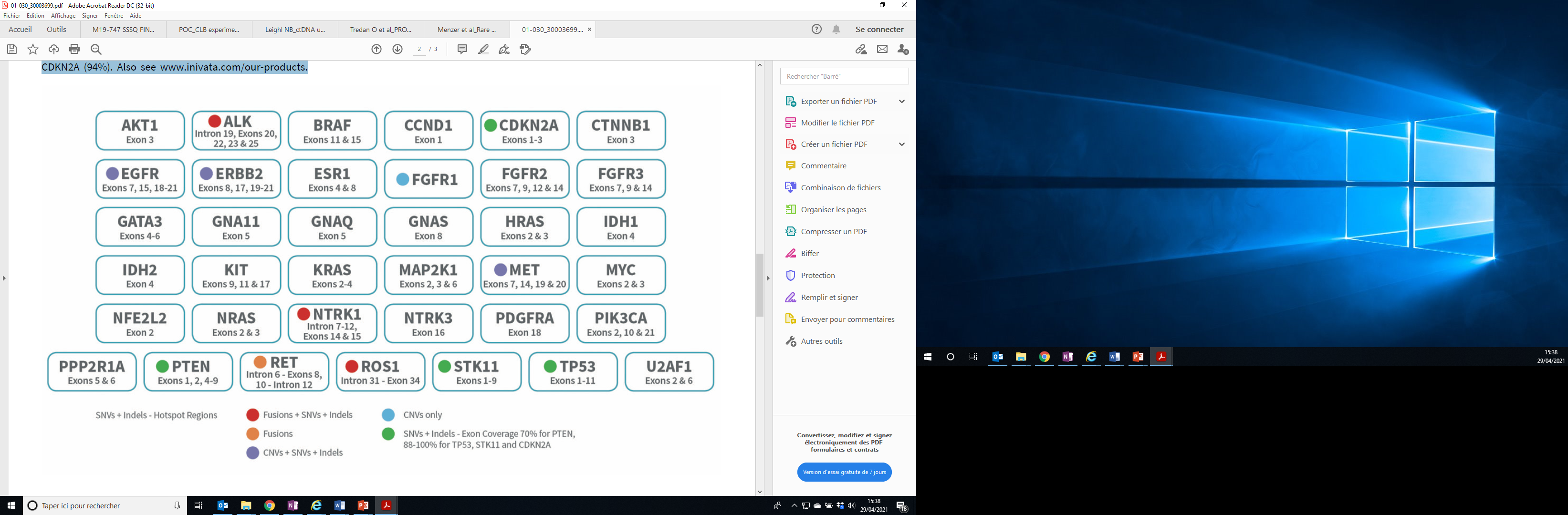


FusionPlexRNA CTL (Archedex):

Detection of somatic mutations: AKT1 (3) , ALK (21 à 25), AXL (5, 11, 15, 17), BRAF (11, 15) , CTNNB1 (3), DDR2 (17) , EGFR (18, 19, 20, 21), ERBB2 (20), FGFR2 (8*), FGFR3 (7, 9*, 10*), GNAS (8, 9), HRAS (2, 3, 4), IDH1 (4), IDH2 (4), KIT (11, 13, 17), KRAS (2, 3, 4), MAP2K1 (2, 3) , MET (14 à 20), NRAS (2, 3, 4), POLE (9 à 14), PIK3CA (9, 20), RET (11, 13, 14, 15, 16), ROS1 (38)

Detection of fusion transcripts: ALK, AXL, BRAF, CCND1, FGFR1, FGFR2, FGFR3, MET, NRG1, NTRK1, NTRK2, NTRK3, PPARG, RAF1, RET, ROS1

Detection of overexpression: ALK, CCND1, EGFR, ERBB2, FGFR1, FGFR2, FGFR3, KIT, MET, NTRK1, NTRK2, NTRK3, RET, ROS1
